# Case Report: A Rare Case of Severe Coronary Venous Spasm During Radiofrequency Ablation of Premature Ventricular Contraction

**DOI:** 10.3389/fcvm.2022.889761

**Published:** 2022-05-02

**Authors:** Rina Sha, Bing Rong, Kellina Maduray, Jingquan Zhong

**Affiliations:** ^1^The Key Laboratory of Cardiovascular Remodeling and Function Research, Chinese Ministry of Education, Chinese National Health Commission and Chinese Academy of Medical Sciences, The State and Shandong Province Joint Key Laboratory of Translational Cardiovascular Medicine, Department of Cardiology, Qilu Hospital, Cheeloo College of Medicine, Shandong University, Jinan, China; ^2^Department of Cardiology, Qilu Hospital (Qingdao), Cheeloo College of Medicine, Shandong University, Qingdao, China

**Keywords:** coronary venous spasm, premature ventricular contraction, radiofrequency ablation, mechanism, coronary artery spasm

## Abstract

Coronary venous spasm has never been reported during premature ventricular extrasystole ablation. We report a 20-year-old female patient who experienced a severe spasm of the great cardiac vein during radiofrequency ablation for premature ventricular contractions, which were relieved eventually by the administration of intracoronary nitroglycerine. The operation was successfully completed, leading to a long-term resolution of her palpitation symptoms.

## Introduction

The great cardiac vein is often used as a target site for radiofrequency ablation (RFA) when treating premature ventricular contraction (PVC) in an atypical site (1). In previous reports, RFA was associated with a rare but serious side effect, coronary artery spasm, whose major possible mechanism may be catheter ablation energy inflicting direct thermal trauma near the coronary artery or impairment of the autonomic nervous system (2–4). However, coronary venous spasm (CVS) is an under-recognized phenomenon. In this report, we describe a case of CVS during the RFA of PVC.

## Case Description

**Figure 1:**
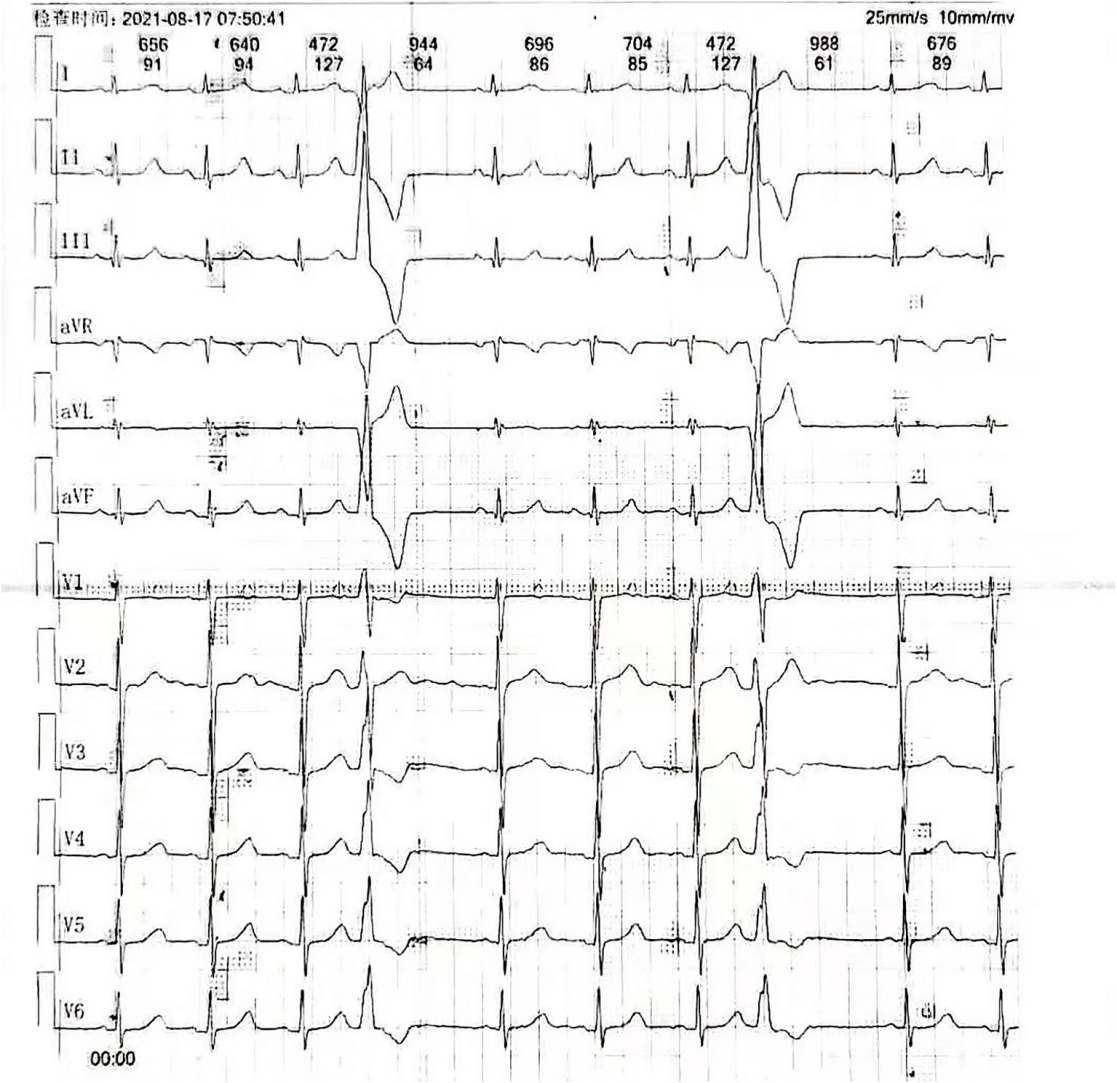
Twelve-lead electrocardiogram displaying premature ventricular contractions.

The patient was a 20-year-old female with a history of PVCs. Her medical history stated she had no known allergies, history of tobacco or alcohol usage, family history of sudden death or cardiac disease, or history of surgery. With the aim of treating her palpitations, she was admitted to Ningjin County PPL's Hospital in Shandong province for RFA. Before the ablation, a 12-lead electrocardiogram showed frequent PVCs (Figure 1). There was no significant abnormity in her transthoracic echocardiogram or biochemical indexes.

**Figure 2:**
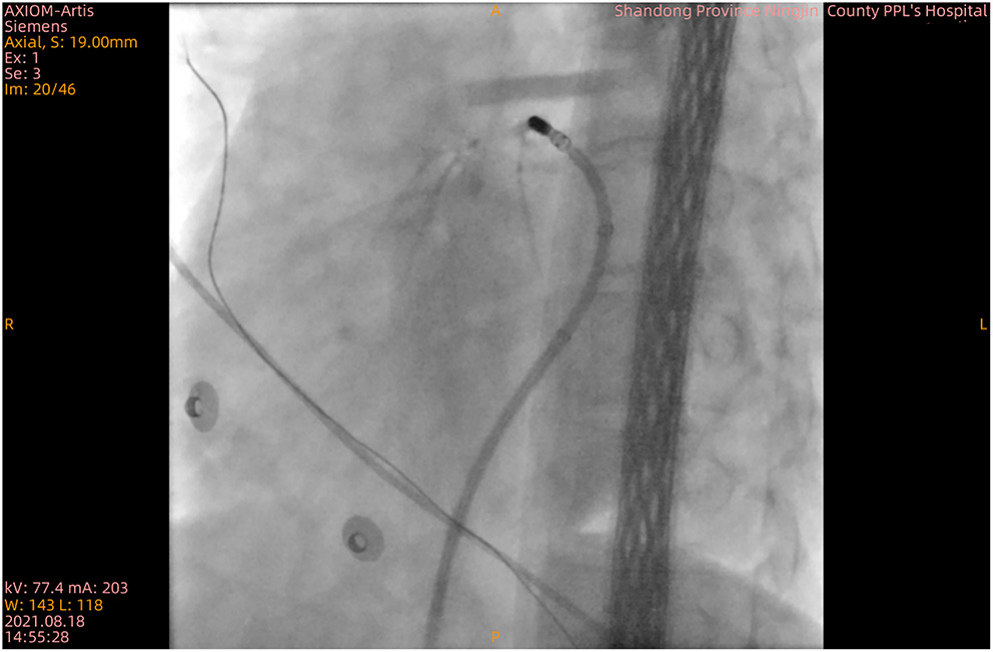
X-ray confirming blood circulation in the great cardiac vein.

After discontinuation of all anti-arrhythmic medications for at least five half-lives, the patient underwent an electrophysiologic study under local anesthesia, and then mapping and ablation were performed with an 8-Fr decapolar catheter (SmartTouch, Biosense Webster, United States). PVCs were demonstrated in the great cardiac vein, and then the catheter was positioned (Figure 2 and Supplementary Video 1). The impedance at the ideal mapping position immediately increased to about 300 Ω, and radiofrequency energy was delivered at a power of 25 W with a saline irrigation flow velocity of 17 ml/min. After 20 s, an X-ray revealed that the catheter was impacted at this position (Supplementary Video 1). After several failed attempts to extract the catheter, the abnormality was considered to be vasospasm. A venogram was immediately manipulated to confirm great cardiac vein flow and rule out cardiac tamponade (Figure 2). Saline was simultaneously injected at a rate of 1 ml/min *via* the intracoronary vein. The angiography showed pericardial effusion. Pericardiocentesis was performed by extracting a 40 ml colorless transparent liquid, which appeared to be saline that was effused from the catheter. Approximately, 4 min after intracoronary injection of nitroglycerin (200 ug), the vasoconstriction was rapidly relieved (Supplementary Video 2). An angiogram *via* the coronary sinus was performed, which showed no signs of exudation, suggesting effusion of pericardial fluid from the catheter but not perforation. Mapping and ablation were continued, with vasoconstriction ceased to persist, and the patient ultimately converted to sinus rhythm twice, each time with 60 s. The procedure was finalized uneventfully.

## Discussion

The differential diagnosis includes (1) venous thrombosis, which is not consistent with the X-ray results, (2) local hematoma resulting in an impacted catheter, which was excluded considering that the venogram showed blood flow in the great cardiac vein, (3) angioedema caused by ablation energy, which was unlikely, since there were no associated signs on X-ray, and (4) severe venous spasm was the maximum possibility since it can be alleviated by vasodilation, in this case, with nitroglycerin.

As mentioned above, coronary artery spasm has been well-documented. Peripheral venous spasm (5), central venous spasm during pacemaker implantation (6–8), and saphenous venous graft spasm causing recurrent angina (9) have been previously reported. CVS has never been reported in the literature. The pathophysiological explanation may be the same as arterial spasm.

Vasospasm could be induced by certain stimulants, such as mechanical stimulation, nerve stimulation, platelet dysfunction, and vasoconstrictor substances (9, 10). During ablation, simple mechanical stimulation to the venous vascular smooth muscle layer with a large ablation catheter could cause an imbalance between vasoconstrictors and vasodilators, resulting in vasospasm. It could also be associated with endothelial dysfunction as a consequence of direct thermal damage, since a healthy intact endothelium may prevent vasoconstriction by releasing endothelium-derived relaxing factors. Moreover, considering the great cardiac vein is surrounded by epicardial adipose tissue, which produces a large amount of metabolically active substances with both endocrine and paracrine actions, the vasospasm may have been the result of an injured adipocyte tissue (11). However, we are uncertain whether the great cardiac vein or the epicardial adipose tissue was injured through direct thermal trauma. We suggest that the most likely cause of the CVS was chemical autoregulatory imbalance. However, the definitive mechanism remains undetermined and requires further studies. Regarding precautionary measures, the operator should focus on impedance. If it reaches 300 Ω, radiofrequency ablation should be stopped. After impedance is reduced to a normal range with saline irrigation, the operator may proceed.

## Limitations

The activity of the vasoconstrictors and vasodilators in the great cardiac vein during an ablation procedure could not be directly confirmed. Therefore, we were unable to ensure a cause-and-effect relationship in this case.

## Conclusion

We reported a rare clinical case of CVS during PVC ablation, which was alleviated by intracoronary infusion of nitroglycerin. We suspect that the vasospasm was induced by the application of catheter ablation energy and catheterization in the great cardiac vein, which may result in an imbalance between vasoconstrictors and vasodilators.

## Data Availability Statement

The raw data supporting the conclusions of this article will be made available by the authors, without undue reservation.

## Ethics Statement

The studies involving human participants were reviewed and approved by Ethics Committee of Shandong University Qilu Hospital. The patients/participants provided their written informed consent to participate in this study. Written informed consent was obtained from the individual(s) for the publication of any potentially identifiable images or data included in this article.

## Author Contributions

RS and BR contributed to the clinical treatment of this case. RS and KM contributed to the writing of the manuscript. JZ contributed to the review of the manuscript. All the authors listed have contributed sufficiently to the project in order to be included as authors and approved the final version of the manuscript for publication.

## Conflict of Interest

The authors declare that the research was conducted in the absence of any commercial or financial relationships that could be construed as a potential conflict of interest.

## Publisher's Note

All claims expressed in this article are solely those of the authors and do not necessarily represent those of their affiliated organizations, or those of the publisher, the editors and the reviewers. Any product that may be evaluated in this article, or claim that may be made by its manufacturer, is not guaranteed or endorsed by the publisher.

## Supplementary Material

Supplementary Video 1 X-ray showing the vasospasm and an impacted catheter at the site of the great cardiac vein.

Supplementary Video 2 X-ray showing that the vasospasm was rapidly relieved.

## References

[1] Li T Xu Q Zhan XZ Xue YM Liao HT Li YF Letsas KP. Unique electrocardiographic pattern “w” wave in lead I of idiopathic ventricular arrhythmias arising from the distal great cardiac vein. BMC Cardiovasc Disord. (2019) 19:90. 10.1186/s12872-019-1064-9 30987582 PMC6466655

[2] Yamashita E Tada H Tadokoro K Hashimoto T Kaseno K Miyaji K. Left atrial catheter ablation promotes vasoconstriction of the right coronary artery. Pacing Clin Electrophysiol. (2007) 30:S98–102. 10.1111/j.1540-8159.2007.00615.x 17302728

[3] Tada H Naito S Oshima S Taniguchi K. Vasospastic angina shortly after left atrial catheter ablation for atrial fibrillation. Heart Rhythm. (2005) 2:867–70. 10.1016/j.hrthm.2005.05.009 16051126

[4] Honda N Takase S Tashiro H. Severe coronary artery spasm repeatedly induced after left pulmonary vein isolation in patient with atrial fibrillation. HeartRhythm Case Rep. (2018) 4:501–5. 10.1016/j.hrcr.2018.07.010 30479946 PMC6241036

[5] Wennevold A Christiansen I Lindeneg O. Complications in 4,413 catheterizations of the right side of the heart. Am Heart J. (1965) 69:173–80. 10.1016/0002-8703(65)90034-7 14256692

[6] Cooper RM Krishnan U Pyatt JR. Central venous spasm during pacemaker insertion. Heart. (2010) 96:1484. 10.1136/hrt.2010.203919 20813728

[7] Clemens RK Lillis AP Alomari AI. Catheter-induced venous spasm. Circulation. (2012) 126:2363–5. 10.1161/CIRCULATIONAHA.112.112946 23129703

[8] Vemuri KS Parashar N Bootla D Revaiah PC Kanabar K Nevali KP. Refractory axillary venous spasm during permanent pacemaker implantation. Egypt Heart J. (2020) 72:71. 10.1186/s43044-020-00102-z 33079321 PMC7575655

[9] Victor MF Kimbiris D Iskandrian AS Mintz GS Bemis CE Procacci PM. Spasm of a saphenous vein bypass graft. A possible mechanism for occlusion of the venous graft. Chest. (1981) 80:413–5. 10.1378/chest.80.4.413 6974087

[10] He GW Rosenfeldt FL Buxton BF Angus JA. Reactivity of human isolated internal mammary artery to constrictor and dilator agents. Implications for treatment of internal mammary artery spasm. Circulation. (1989) 80:I141–150. 2766521

[11] Villasante Fricke AC Iacobellis G. Epicardial adipose tissue: clinical biomarker of cardio-metabolic risk. Int J Mol Sci. (2019) 20. 10.3390/ijms20235989 31795098 PMC6929015

